# Assessment of preoperative planning and intraoperative accuracy of the AIKNEE system for total knee arthroplasty

**DOI:** 10.1186/s12891-024-07645-1

**Published:** 2024-07-19

**Authors:** Saijiao Lan, Jian Li

**Affiliations:** 1 Department of Sports Medicine, Fujian Province Second People’s Hospital, No.282, Wusi Road, Gu Lou District, Fuzhou, 350001 Fujian China; 2 Department of Sports Medicine, The Second Affiliated Hospital of Fujian University of Traditional Chinese Medicine, Fuzhou, 350001 Fujian China

**Keywords:** Total knee arthroplasty, AIKNEE system, Preoperative planning, Intraoperative accuracy, Postoperative outcomes

## Abstract

**Background:**

The aim of this retrospective study was to evaluate the effectiveness and accuracy of the AIKNEE system in preoperative planning and intraoperative alignment for total knee arthroplasty (TKA).

**Methods:**

A total of 64 patients were planned preoperatively by the AIKNEE system, including the measurement of mechanical femorotibial angle (mFTA), lateral distal femoral angle (LDFA), and medial proximal tibial angle (MPTA) using three-dimensional reconstructed images. Intraoperatively, the actual prosthesis size and alignment were compared to the planned parameters. Postoperative outcomes, including pain levels, range of motion (ROM), and Knee Scoring System (KSS) scores, were assessed after surgery. Statistical analyses were performed to evaluate the correlation between alignment deviations and postoperative function.

**Results:**

The AIKNEE system accurately predicted the prosthesis size in thirty-one of femoral cases (48%) and forty-seven of tibial cases (73%). Deviations of mFTA, LDFA, and MPTA from the target value were within 3° in 88%, 92%, and 95% of cases, respectively. A significant improvement was observed in postoperative pain, ROM, and KSS scores (*p* < 0.001). Correlation analysis revealed that greater deviations in mFTA and LDFA were associated with increased pain (*p* = 0.004, 0.047) and lower KSS scores (*p* = 0.027).

**Conclusion:**

The AIKNEE system demonstrated promising results in predicting prosthesis size and achieved alignment within the desired range in a majority of cases. Postoperative outcomes, including pain levels and functional improvement, were favorable.

## Introduction

The preoperative planning of precise is advantageous for promoting knee prosthesis alignment, minimizing postoperative pain, and enhancing patient satisfaction [1–4]. X-ray and computed tomography (CT) imaging techniques offer crucial data for preoperative planning. While X-ray imaging is economical and convenient, it necessitates a high level of expertise from the radiologists. On the other hand, CT imaging is not constrained by the patient’s position and imaging proficiency, and can be directly reconstructed using software to facilitate preoperative planning [5, 6]. Prediction of prosthesis sizes derived three-dimensional (3D) reconstructed knees are more sensitive and accurate than results from two-dimensional (2D) images [7].

In a clinical setting, 3D reconstruction of CT images helps formulate more reasonable surgical planning. Especially, it was combined with artificial intelligence, such as PSI devices and robotic-assisted, which are costly [7]. Intelligent autonomous planning is the further development of artificial intelligence technology, is the ultimate goal of mission planning, and also economical, convenient. Traditionally, Mimics software is commonly used for preoperative planning and require image segmentation, which is time-consuming and prone to human error [8]. CT Images are acquired and imported into the AIKNEE system, and a report is created. The report covers knee parameters, osteotomy thickness, and prosthesis sizes. At present, the clinical application of AIKNEE system is still rare, and its accuracy is still uncertain; Therefore, the study was to evaluate the effectiveness and accuracy of the AIKNEE system in preoperative planning and intraoperative alignment for total knee arthroplasty (TKA).

## Materials and methods

### Patients

This was an institutional review board-approved retrospective study (Fujian Province Second People’s Hospital, SPHFJP-K2022017-02). Patients who had perfected preoperative planning for AIKNEE and underwent total knee arthroplasty between January 2021 and October 2022 were included. All procedures were performed by a fellowship-trained orthopedic surgeon at an academic institution.

### AIKNEE Workflow

**Fig. 1:**
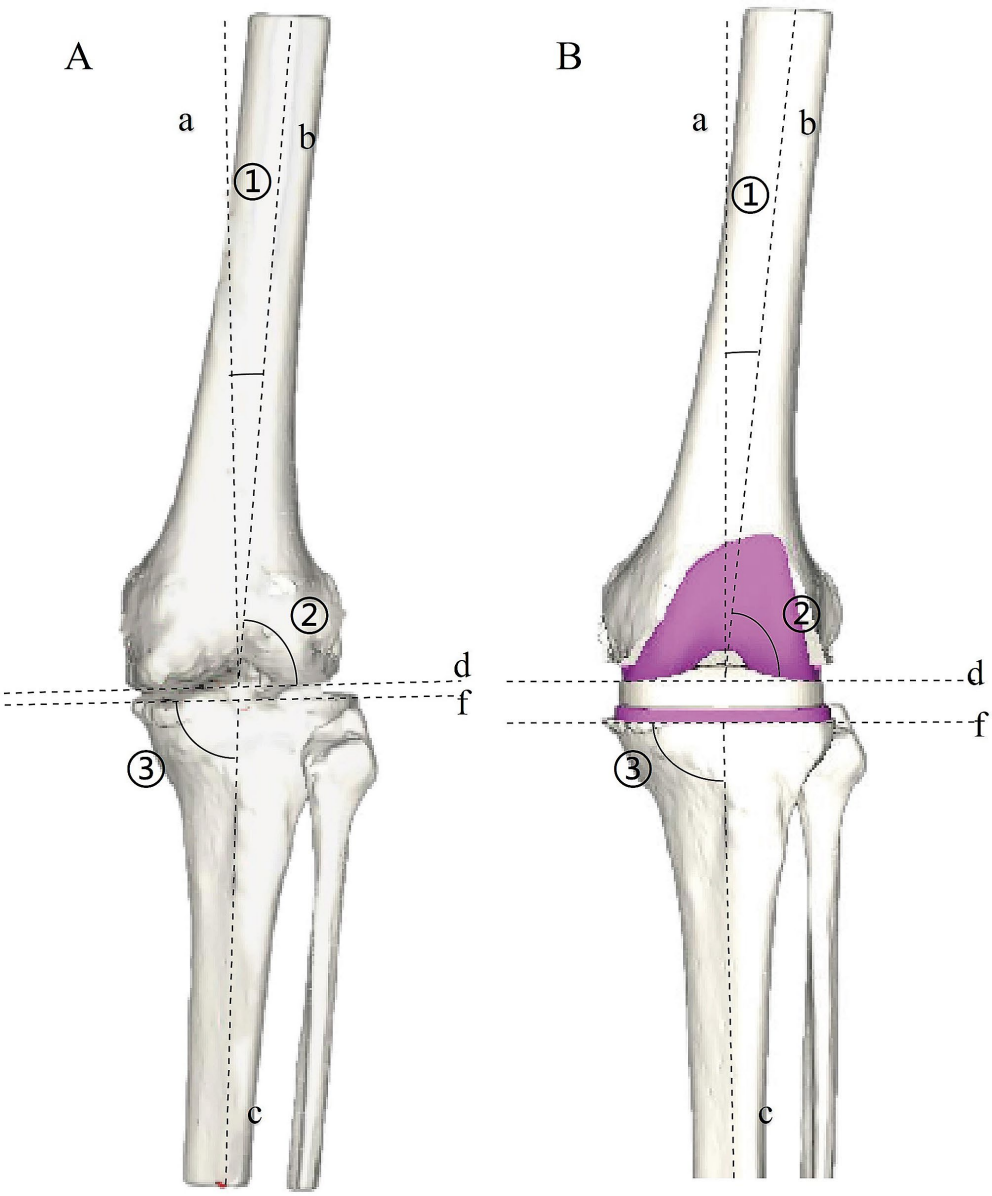
(**A**) AIKNEE system preoperative planning imaging; (**B**) AIKNEE system Post-planning imaging, Pink means the location of the planned prosthesis; Line a: mechanical axis of femur; line b: anatomical axis of femur; line c: mechanical axis of tibia; line d: parallel line of distal femur; line f: tibial plateau line; mFTA: the angle between line a and line c; ①: anatomic mechanic axis deviation (AMA), the angle between line a and line b; ②: lateral distal femoral angle (LDFA), the lateral angle between line a and line d; ③: medial proximal tibial angle (MPTA), the angle between line c and line f

1. Import DICOM images and reconstruct the knee joint in three-dimensions.
2. The AIKNEE system automatically identifies and labels the mechanical axis of the femur (line a), the anatomical axis of the femur (line b) and the mechanical axis of the tibia (line c) on the 3D reconstructed image (Fig. 1A). Measurement of angles such as mechanical femorotibial angle (mFTA), anatomical femorotibial angle (aFTA), anatomic mechanic axis deviation (AMA), posterior condylar axis (PCA), the lateral distal femoral angle (LDFA), the medial proximal tibial angle (MPTA), and joint line convergence angle (JLCA).
3. The AIKNEE system further analyzes the measured data to estimate the thickness of the femoral and tibial osteotomies, predict the size of the prosthesis, and simulate its placement (Fig. 1B). Review of mechanical axis alignment again (line a perpendicular to line d, line c perpendicular to line f and the angle between line a and line c is 180°), observe the fit of the prosthesis and generate a preoperative planning report. The surgeon refers to this report to draw up the surgical plan and for intraoperative reference.

### Knee-related parameters

mFTA: the angle between line a and line c, is a direct indicator of the observed alignment of the mechanical axis, which is maintained with a target of 180°; AMA: The angle between line a and line b (Fig. 1A) is an important indicator of the presence of extra-articular deformity, with a normal range of 5° to 7°. JLCA: the angle between line d and line f, to evaluate the gap balance, the target is 0°, meaning that parallelism between lines d and f. LDFA: the lateral angle between line a and line d (Fig. 1A); MPTA: the angle between line c and line f (Fig. 1A); LDFA and MPTA are aimed at 90° to achieve mechanical alignments. Deviation of each angle from the target value, such as mFTA, LDFA and MPTA, within 3° can effectively improve the survival rate of the prosthesis [9–12]. Deviations within 3° were designated as non-outliers in this study, and those beyond 3° as outliers.

### Data collection

1. Preoperative data: patient demographics age, sex, body mass index, Knee Scoring System (KSS) Score, Visual Analogue Score (VAS), range of motion (ROM).

KSS: an important index that responds to knee recovery after total knee arthroplasty [13, 14]. It was used to assess patients’ VAS scores for walking on level ground, stairs or slopes, as well as knee stability, ROM and distance traveled. Knee function was assessed at preoperatively, postoperatively with the use of the KSS Score.

(2) The deviation between the postoperative mFTA, LDFA, MPTA and the surgical target value is marked as a D(Deviation) value, and it can also be considered a degree of residual deformity. Analysis of the correlation between D values and postoperative ROM, VAS scores and KSS scores to observe the effect of D values on postoperative function.
(3) Preoperative planning reports can be referenced before the preparation of prototype sizes. The AIKNEE predictive prosthesis sizes is prioritized. If the predicted size of the prosthesis is perfect match, only one insertions test is required; And more insertions for a deviation of more than 1 size, which is related to the experience of the surgeon. The enhancement of the accuracy rate of preoperative planning of prostheses sizes, decreases operation time and saves surgical resources, reduces the number of intraoperative prosthesis insertions, and promotes functional recovery after surgery [9, 11].

### Statistical analysis

Statistical analyses were performed using the SPSS20.0 software. The measurement data were expressed as mean ± standard deviation (x ± s), and all were in line with approximate normal distribution or normal distribution. Comparisons of indexes before and after surgery within groups were performed using paired samples t-tests. The Chi-square test was used for qualitative values. Knee-related parameters were correlated with postoperative ROM, VAS score and KSS score using spearman correlation analysis. The statistical significance was set at *p* < 0.05 for all tests.

## Results

A total of 64 patients were included in the study, mean age 68.17 ± 7.94 years, Body Mass Index (BMI) 26.52 ± 4.22 kg/m^2^.

According to the results of correlation analysis, there was a negative correlation between the D value and the knee function (*p* < 0.05). The larger the mFTA deviation from target values, the lower the KSS scores, the poorer the knee function. (*p* = 0.027). The severity of pain intensity worse with increasing mFTA or LDFA divergence from target values (*p* = 0.004, 0.047) (Table 1). Additionally, the study suggests a close correlation between alignment, postoperative pain, and function.

In this study, the patients primarily had varus knee deformities; The mechanical axis was corrected from 189.31 ± 5.08° to 181.21 ± 2.40°, and 88% of mFTA, 92% of LDFA, and 95% of MPTA were within 3° of our target values, effectively correcting the alignment deformities in most patients. (Table 2)

The preoperative JLCA of 5.97 ± 3.37° was significantly reduced to 0.31 ± 0.28° postoperatively, resulting in nearly parallel joint lines, achieving gap balancing. (Table 2) Significant improvements were observed in KSS knee score, VAS score, and ROM postoperatively compared to preoperative measurements. (*p* < 0.001) (Table 2).

**Table 1:** Correlation analysis of the postoperative outlier differences of each knee parameter with function

		mFTA^a^	LDFA^b^	MPTA^c^
Activity	r	-0.034	0.062	-0.001
	p	0.790	0.628	0.994
VAS scores	r	0.356	0.249	0.164
	p	0.004^*^	0.047^*^	0.196
KSS scores	r	-0.276	-0.062	-0.025
	p	0.027^*^	0.628	0.844

a: Difference between the postoperative mFTA and 180°;

b: postoperative difference between the postoperative LDFA and 90°;

c: the difference between the postoperative MPTA and 90°;

*: The data were statistically significant, *p* < 0.05;

VAS: visual analogue score;

KSS: knee society system score.

**Table 2:** Preoperative and postoperative patient conditions

	preoperative	postoperative	*P*
mFTA	189.31 ± 5.08*	181.21 ± 2.40	0.000
mFTA^#^ (%)	-	56(88%)	
LDFA^#^ (%)	-	59(92%)	
MPTA^#^ (%)	-	61(95%)	
JLCA	5.97 ± 3.37°*	0.31 ± 0.28°	0.000
ROM	105.31 ± 15.56	116.56 ± 6.42	0.000
VAS	6.14 ± 0.71	0.27 ± 0.48	0.000
KSS	98.66 ± 13.35	148.67 ± 8.20	0.000

*: means that the preoperative mFTA, JLCA is different from the target value by one-sample t-test (*p* < 0.05);

#: Means the case of patient within 3° of our target value for mFTA, LDFA, MPTA;

mFTA: mechanical femorotibial angle;

LDFA: lateral distal femoral angle;

MPTA: medial proximal tibial angle;

JLCA: joint line convergence angle;

VAS: visual analogue score;

KSS: knee society system score.

The accuracy of femoral and tibial prosthesis sizes was 48% and 73%, respectively; The mean intraoperative femoral side prosthetics were inserted 1.67 ± 0.82 times, the tibial side were inserted 1.38 ± 0.70 times; The tibial side prediction accuracy was greater (*p* = 0.004), and the number of prostheses insertions was fewer (*p* = 0.009). (Table 3)

**Table 3:** Statistical analysis of the number of intraoperative prosthesis insertion trials

insertion trials(time)	Femoral side (cases)	Tibial side (cases)	*P*
1	31(48%)	47(73%)	0.004
≤ 2	56(88%)	59(92%)	0.380
>2	8(13%)	5(8%)	0.388

%: Percentage of cases.

In patients with more than 2 inserts of femoral and tibial prosthesis, 92% of patients had flexion contracture deformity; One of the patients had a flexion deformity of 15°, a lax medial collateral ligament, and the largest deviation in tibia and femur model. Consideration of flexion deformity and ligament relaxation may have an impact on its prediction.

### Case report

**Fig. 2:**
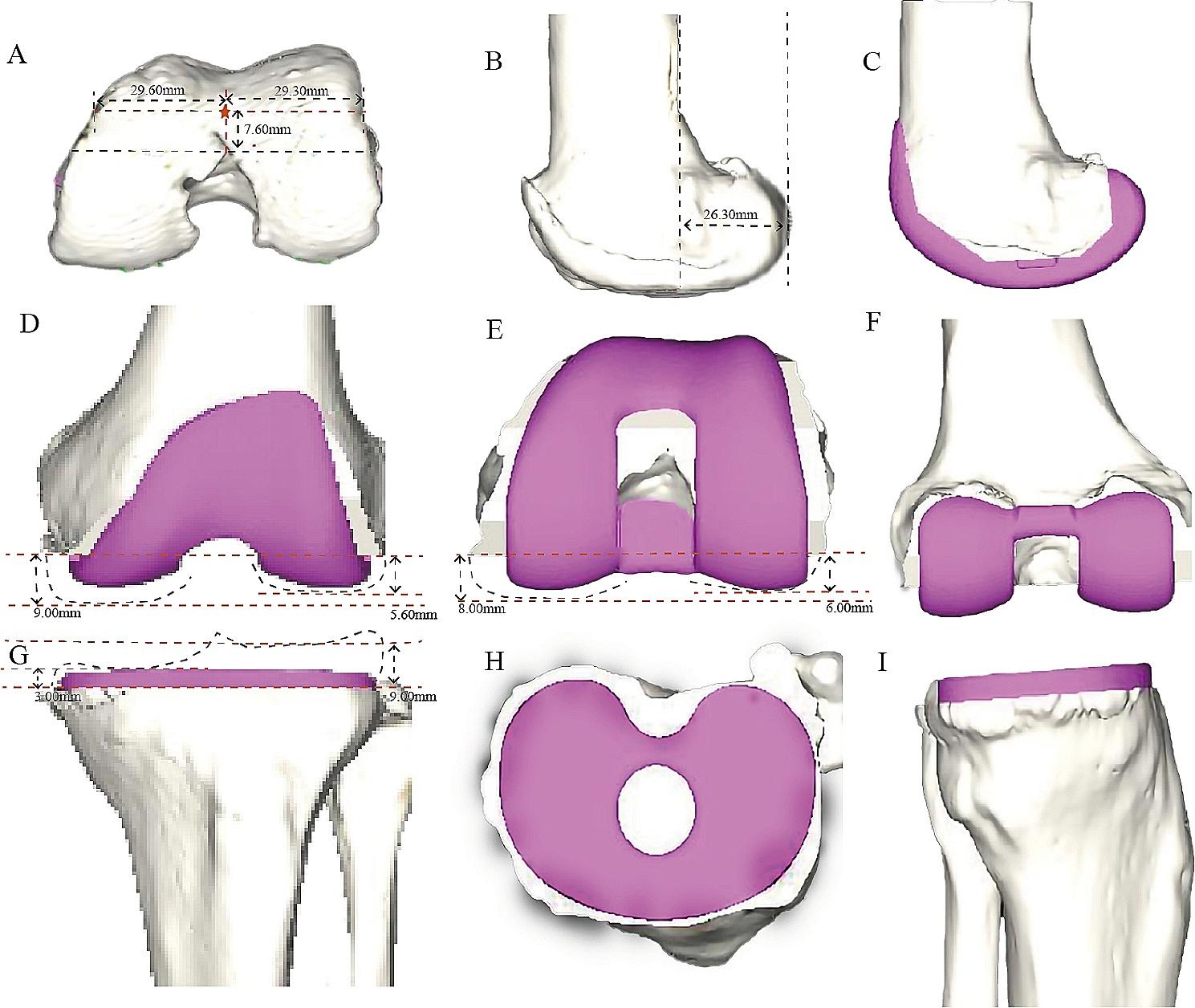
(**A**-**B**) AIKNEE system automatically generates relevant data before prosthesis planning: the position of the pentagram is the point of the femur penetration. (**C**-**D**) AIKNEE system plans for femoral side prostheses: Pink means the location of the planned prosthesis; The gray line means the original internal and external femoral condyles; The black line represents the osteotomy thickness planned. (**G**-**I**) AIKNEE system plans for tibial side prostheses: Pink means the location of the planned prosthesis; The gray line means the original tibial plateau position; The black line means the osteotomy thickness planned

**Fig. 3:**
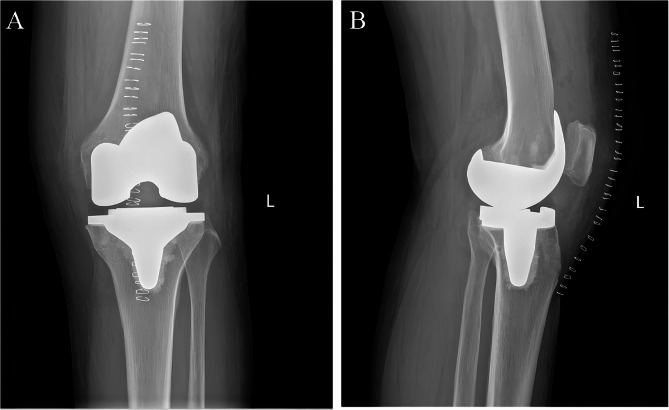
Post operation X-ray (AP and lateral view) of left knee after total knee arthroplasty

A 68-year-old woman admitted to our clinic with left knee pain and limitation in knee flexion. Admission check: The left knee had a flexion contracture of 15°, and the ROM of the left knee was 15° (extension) to 90° (flexion), and instability was observed in the stress tests. The patient with an mFTA of 184.5° and an aFTA of 178.5°, indicative of a patient with an unstable varus deformity. The.

preoperative KSS score was 75. Preoperative planning predicted a prosthesis size of ATTUNE size-3 by the AIKNEE software (Fig. 2). Intraoperatively, it was adjusted to an ATTUNE size-6 prosthesis and the medial collateral ligament was reconstructed. Postoperative review of the left knee X-ray (Fig. 3). The patients started with active and passive ROM exercises from the first postoperative day. Follow-up of the patient was performed at 1-month, 3-months, and 6-months after surgery. Post-surgery, the varus deformity was corrected, and the patient experienced good functional recovery.

## Discussion

Mechanical alignment, gap balance and prosthesis fit are key factors in postoperative pain and functional improvement. This study found a positive correlation between the degree of residual deformity (D value) of the mechanical axis mFTA and LDFA with postoperative pain, indicating that worse coronal alignment of the knee joint leads to greater postoperative pain and poorer function. And gap balancing improves buckling stability [15–17]. However, we cannot overlook the fit of the prosthesis. Looking at the long-term effects, a well-fitted prosthesis reduces the risk of postoperative joint pain and component loosening [4, 8, 18]. The higher the accuracy of the prediction of anatomical positioning, osteotomy thickness, and knee size, the more effective it is to promote alignment, gap balancing, reduce test insertion, simplify surgical steps, and save surgical time [9, 11, 19]. Therefore, whether it’s correcting the alignment, predicting osteotomy thickness, or matching the prosthesis, all are crucial in preoperative planning.

### Preoperative and postoperative functional changes

Understanding joint characteristics is crucial for improving surgical accuracy and prosthesis lifespan. It also serves as a crucial prerequisite for surgery. Because of this, preoperative parameter measurement accuracy is crucial. Compared to X-rays, CT imaging, the location of the patient, the projection angle and the experience of the photographer, are the key tools for osteopathic surgery [20, 21]. Michel P. Bonnin et al. found that CT was more accurate in parameters measurement such as mFTA and more optimal in predicting the size and position of the prosthesis [22–24]. 3D CT reconstruction of the femur and tibia gives the surgeon more accurate preoperative data, prediction of osteotomy thickness and prosthesis size [7, 25]. This information aids in the reconstruction of mechanical alignment and soft tissue balance during surgery, which helps the patient regain function. In this study the mFTA was 189.31 ± 5.08° preoperatively and 181.21 ± 2.40° after intraoperative correction of the valgus deformity (*p* < 0.001). The preoperative JLCA of 5.97 ± 3.37° was significantly reduced to 0.31 ± 0.28° postoperatively, resulting in nearly parallel joint lines, achieving gap balancing, improving buckling stability. The KSS score for knee function improved from 98.66 ± 13.35 to 148.67 ± 8.20, pain was reduced from 6.14 ± 0.71 to 0.27 ± 0.48, and ROM improved significantly. The quality of patient survival was improved (*p* < 0.001).

### Correlation analysis of knee joint parameter outliers

Results of this study indicate that the mechanical axes of the lower limbs, represented by the D value of mFTA, correlated with pain and knee function. The D value of LDFA is associated with increased severity of pain. And the greater the D value, the greater deviation from target value, the worse pain. The alignment of the mechanical axis of the femur and tibia in relation to the osteotomy plane has a direct impact on the pressure distribution in the double compartment, which is closely associated with postoperative knee pain and functional activities [26–28]. Furthermore, a significant proportion of the mFTA, LDFA, and MPTA measurements (88%, 92%, and 95% respectively) achieved the desired D value of less than 3°. Observation results of Michel P. Bonnin et al. showed that the Knee-Plan software (Symbios, Yverdon les bains, Switzerland) was utilized for preoperative planning, resulting in 84% of the D values for mFTA, LDFA, and MPTA being less than 3°, which is comparable to the findings of our study [23, 29, 30].

### Analysis of the fit of the knee sizes

Poor fit is often associated with complications such as loosening of the prosthesis and functional impairment [5, 8, 18]. Although using X-ray images to establish a two-dimensional model is quick and cheap, it cannot effectively forecast the trend of knee alignment in patients [5, 31]. The three-dimensional model demonstrates a high level of accuracy in predicting the actual implant size, with some studies reporting accuracy rates of up to 100%. In contrast, the two-dimensional digital model only predicts the actual implant size in approximately 50% of cases [7]. The AIKNEE system exhibits an accuracy rate of 48% for femoral prostheses and 73% for tibial prostheses. Notably, the accuracy on the tibial side is significantly higher than that on the femoral side (*p* = 0.004). Additionally, the insertion of the tibial side prosthesis is less frequent (*p* = 0.009), and the accuracy of the tibial side prediction surpasses that of the two-dimensional model. In comparison to Miura M.D. et al. the utilization of the MIMICS system for the prediction of femoral and tibial components prostheses yielded an accuracy of 44.3% and 57.00% respectively [8]. Conversely, the AIKNEE system demonstrated a significantly enhanced accuracy for tibial prostheses, while also offering the advantages of reduced time and simplified operation when compared to Mimics software, with an average time per case reduced from approximately 24 min to only about 5 min [32–34]. However, as indicated by Vicente J et al., the accuracy of tibial prostheses prediction reached 92%, while femoral prostheses prediction exceeded 95%, thus displaying a high level of consistency [35]. Kotela et al. discovered that MyKnee achieved an 89.8% accuracy rate in predicting femoral components and 75.8% accuracy rate in predicting tibial components [36]. In comparison to the MyKnee system, the AIKNEE system’s accuracy requires further enhancement, and overall, its prosthesis prediction level is considered moderate.

### Limitations

In this study, it is worth noting that one patient exhibited a flexion deformity of 15° and laxity in the medial collateral ligament, which resulted in the largest deviation in tibia and femur size, as well as the highest number of insertions. The failure to exclude factors that might have affected its accuracy was a weakness of this trial. Factors affecting accuracy include severe coronary deformity > 15°, knee stiffness and inadequate extension > 15°, flexion range < 90°, severe medial relaxation > 10°, or severe lateral relaxation > 15° [23]. Furthermore, the absence of cartilage visibility on CT scans may introduce bias in predicting osteotomy thickness, and the precision of preoperative planning may diminish in cases of trochlear dysplasia [37].

## Conclusion

The AIKNEE system demonstrated promising results in predicting prosthesis size and achieved alignment within the desired range in a majority of cases. Postoperative outcomes, including pain levels and functional improvement, were favorable.

## Data Availability

All data are fully available without restriction. Requests for data can be made to the corresponding author.

## Abbreviations

TKA: total knee arthroplasty
mFTA: mechanical femorotibial angle
LDFA: lateral distal femoral angle
MPTA: medial proximal tibial angle
aFTA: anatomical femorotibial angle
AMA: anatomic mechanic axis deviation
PCA: posterior condylar axis
3D: three-dimensional
2D: two-dimensional
ROM: range of motion
VAS: visual analogue score
KSS: knee scoring system scores
JLCA: joint line convergence angle
D: Deviation
